# Donor metabolic characteristics drive effects of faecal microbiota transplantation on recipient insulin sensitivity, energy expenditure and intestinal transit time

**DOI:** 10.1136/gutjnl-2019-318320

**Published:** 2019-05-30

**Authors:** Pieter de Groot, Torsten Scheithauer, Guido J Bakker, Andrei Prodan, Evgeni Levin, Muhammad Tanweer Khan, Hilde Herrema, Mariette Ackermans, Mireille J M Serlie, Maurits de Brauw, Johannes H M Levels, Amber Sales, Victor E Gerdes, Marcus Ståhlman, Alinda W M Schimmel, Geesje Dallinga-Thie, Jacques JGHM Bergman, Frits Holleman, Joost B L Hoekstra, Albert Groen, Fredrik Bäckhed, Max Nieuwdorp

**Affiliations:** 1 Department of Internal and Vascular Medicine, Amsterdam University Medical Centres, Amsterdam, The Netherlands; 2 Wallenberg Laboratory, Department of Molecular and Clinical Medicine, Sahlgrenska Academy, Goteborgs Universitet, Gothenburg, Sweden; 3 Department of Surgery, Spaarne Gasthuis, Haarlem, The Netherlands; 4 Department of Gastroenterology, Amsterdam University Medical Centres, Amsterdam, The Netherlands

**Keywords:** diabetes mellitus, intestinal microbiology, gastrointestinal transit, bile acid metabolism

## Abstract

**Objective:**

Bariatric surgery improves glucose metabolism. Recent data suggest that faecal microbiota transplantation (FMT) using faeces from postbariatric surgery diet-induced obese mice in germ-free mice improves glucose metabolism and intestinal homeostasis. We here investigated whether allogenic FMT using faeces from post-Roux-en-Y gastric bypass donors (RYGB-D) compared with using faeces from metabolic syndrome donors (METS-D) has short-term effects on glucose metabolism, intestinal transit time and adipose tissue inflammation in treatment-naïve, obese, insulin-resistant male subjects.

**Design:**

Subjects with metabolic syndrome (n=22) received allogenic FMT either from RYGB-D or METS-D. Hepatic and peripheral insulin sensitivity as well as lipolysis were measured at baseline and 2 weeks after FMT by hyperinsulinaemic euglycaemic stable isotope (^2^H_2_-glucose and ^2^H_5_-glycerol) clamp. Secondary outcome parameters were changes in resting energy expenditure, intestinal transit time, faecal short-chain fatty acids (SCFA) and bile acids, and inflammatory markers in subcutaneous adipose tissue related to intestinal microbiota composition. Faecal SCFA, bile acids, glycaemic control and inflammatory parameters were also evaluated at 8 weeks.

**Results:**

We observed a significant decrease in insulin sensitivity 2 weeks after allogenic METS-D FMT (median rate of glucose disappearance: from 40.6 to 34.0 µmol/kg/min; p<0.01). Moreover, a trend (p=0.052) towards faster intestinal transit time following RYGB-D FMT was seen. Finally, we observed changes in faecal bile acids (increased lithocholic, deoxycholic and (iso)lithocholic acid after METS-D FMT), inflammatory markers (decreased adipose tissue chemokine ligand 2 (CCL2) gene expression and plasma CCL2 after RYGB-D FMT) and changes in several intestinal microbiota taxa.

**Conclusion:**

Allogenic FMT using METS-D decreases insulin sensitivity in metabolic syndrome recipients when compared with using post-RYGB-D. Further research is needed to delineate the role of donor characteristics in FMT efficacy in human insulin-resistant subjects.

**Trial registration number:**

NTR4327.

**Significance of this study**

**What is already known on this subject?**

- Gut microbiota is involved in human health and disease.
- Changes in faecal microbiota are associated with human benign or malign metabolic traits.
- Animal studies have suggested that intestinal transit time and bile acid metabolism are involved in these traits.

**What are the new findings?**

- Faecal microbiota transplantation (FMT) from metabolically compromised obese donors temporarily worsens insulin sensitivity in human metabolic syndrome recipients, whereas a non-significant increase in insulin sensitivity is observed in recipients of FMT from healthy postgastric bypass donors.
- These differential changes are accompanied by alterations in intestinal transit time, resting energy expenditure, plasma metabolites and faecal bile acid composition.
- Response to donor FMT is associated with differences in faecal microbiota composition.

**How might it impact on clinical practice in the foreseeable future?**

- This study helps to quantify the magnitude of gut microbiota-driven metabolic effects after Roux-en-Y gastric bypass.
- This study underscores that poor donor metabolic profile can be transferred by FMT and therefore calls for careful metabolic profiling of faecal donors in and outside of clinical trials to reduce potential harm and increase potential benefit.

## Introduction

Metabolic syndrome (METS) and type 2 diabetes (T2D) are major global health problems for which current pharmacological treatment does not halt disease progression.^1^ Bariatric surgery, in particular Roux-en-Y gastric bypass (RYGB) and vertical sleeve gastrectomy, has been effective in reversing insulin resistance, although the underlying mechanisms are not fully understood. In this regard, data derived from animal models have suggested that metabolic improvements after RYGB may be causally related to an altered gut microbiota composition.^2^

Several studies have shown associations between obesity, intestinal microbiome^3–7^ and decreased intestinal transit time.^8^ Moreover, it has been shown that subjects with normal, impaired and severely impaired (T2D) glucose tolerance can be classified based on their gut microbiota composition.^9^ Despite a multitude of association studies, a causal role for the intestinal microbiota in human disorders of glucose metabolism is less established. We recently confirmed potential causality of intestinal microbiota composition in regulation of human glucose metabolism by showing that faecal microbiota transplantation (FMT) from lean healthy donors improves (peripheral) insulin sensitivity of subjects with METS. The effect was transient and seemed driven by recipient baseline faecal microbiota signature.^10 11^

**Figure 1:**
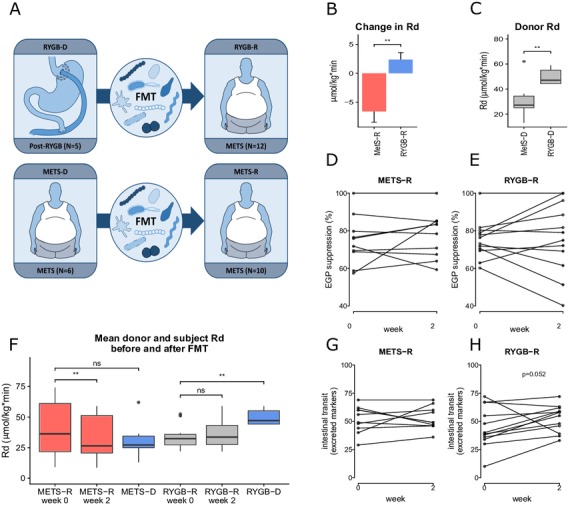
(A) Graphical representation of the study. Five post-RYGB subjects (RYGB-D) donated to 12 subjects with METS (RYGB-R). Six subjects with METS (METS-D) donated to 10 subjects with METS (METS-R). (B) Change in peripheral insulin sensitivity (Rd) between groups is shown as mean±SEM(C) donor Rd and (D and E) hepatic insulin sensitivity (per cent suppression of EGP). (F) Rd in each group before and after FMT and donor Rd at baseline are reported as mean±SEM. For comparison of recipients and donors at baseline (ie, METS-R week 0 vs METS-D and RYGB-R week 0 and RYGB-D) and also for comparison of week 0 vs week 2 for both groups, a paired Wilcoxon rank-sum test was used. (G and H) Change in intestinal transit time, expressed as the number of excreted markers on day 4 after ingestion. EGP, endogenous glucose production; FMT, faecal microbiota transplantation; METS, metabolic syndrome; METS-D, metabolic syndrome donor; METS-R, metabolic syndrome recipient; Rd, rate of glucose disappearance; RYGB-D, Roux-en-Y gastric bypass donor; RYGB-R, Roux-en-Y gastric bypass recipient.   **p≤0.01; NS, not significant (p>0.05).

A pivotal question in our pursuit to assess causality is whether FMT from non-lean donors or from post-RYBG donors (in METS recipients) could also affect glucose metabolism. In support of such an hypothesis, an elegant study by Liou *et al* 2 in diet-induced obesity (DIO) mice showed that FMT from postbariatric surgery DIO donors resulted in significant weight loss and improved metabolism in the DIO recipients. Similarly, FMT from humans following RYGB also produced improved metabolic outcome compared with METS.^12^ We thus repeated this study in humans and investigated short-term effects of allogenic FMT using post-RYGB donors (RYGB-D) or metabolic syndrome donors (METS-D) on insulin sensitivity and lipolysis in METS recipients (RYGB-R and METS-R, respectively; figure 1A), and correlated these effects with changes in gut microbiota composition. Secondary outcome parameters were intestinal transit time adipose tissue inflammatory gene expression, bile acid and short-chain fatty acid (SCFA) metabolism and resting energy expenditure (REE). We hypothesised that RYGB-D FMT leads to increased insulin sensitivity, in conjunction with faster intestinal transit time, as this would provide less opportunity for the bacterial translocation associated with inflammation and insulin resistance.

## Materials and methods

### Study design and inclusion of subjects and donors

Male adult (age 21–69) omnivorous Caucasian obese (body mass index (BMI) ≥30 kg/m^2^) subjects were recruited by newspaper advertisement and screened for METS using the National Cholesterol Education Program (NCEP) criteria for METS (≥3/5: fasting plasma glucose ≥5.6 mmol/L, triglycerides ≥1.7 mmol/L, waist circumference >102 cm, high-density lipoprotein cholesterol <1.03 mmol/L, blood pressure ≥130/85 mm Hg). Inclusion was limited to male Caucasian subjects to increase homogeneity of our study population and reproducibility and comparability across studies. Exclusion criteria were recent weight loss, cardiovascular events, cholecystectomy, use of any systemically acting medication in the last 3 months, a vegetarian diet and use of probiotics. Participants did not take food supplements.

Five post-RYGB-D were selected and recruited by their treating physician at Bariatric Surgery Clinic, Spaarne Hospital, Haarlem, The Netherlands. They were omnivorous, healthy Caucasian men who had lost at least 30% of their presurgery weight after 1 year and did not use any medication (except vitamins). Of note, none of the RYGB-D currently met the NCEP criteria for METS. Donors could donate to multiple recipients. Online supplementary tables S1 and S2 provide an overview of each donor and their respective recipients. METS-D (n=6) were recruited through newspaper advertisement. All donors completed questionnaires regarding dietary and bowel habits, travel history, comorbidity including (family history of) diabetes mellitus and medication use. Donors did not use any medication (including probiotics). Donors were screened for the presence of infectious and other diseases, as specified in the online supplementary methods. Subjects with METS (n=22) were randomised using block randomisation to receive FMT from either RYGB-D or METS-D. Participants were blinded to the allocation for the duration of the study. The primary outcome parameter was change in insulin sensitivity measured by hyperinsulinaemic euglycaemic clamp with stable isotope tracers (^2^H_2_-glucose) after 2 weeks.

10.1136/gutjnl-2019-318320.supp3 Supplementary data

At baseline and 2 weeks after treatment, all studies (including clamp, gastroscopy, 24-hour faeces collection and intestinal transit time measurement) were performed, while at 8 weeks only diet lists, clinical evaluation, and blood and morning faecal collection took place. Participants filled in online dietary intake lists (www.voedingscentrum.nl) for 7 days before each visit to monitor dietary caloric intake and food composition.

Written informed consent was obtained. The study was registered at the Dutch Trial Register (NTR 4327).

### Faecal transplant procedure

No antibiotics were given prior to FMT. A single FMT was performed in each subject. On the day of treatment, the donor produced fresh morning stool at home, which was brought to the hospital by the donor. After admission of the study subject, a duodenal tube was placed by gastroscopy. Each subject then underwent complete colon lavage with 3–4 L of Klean-Prep (macrogol) by duodenal tube until the researcher judged that the bowel was properly lavaged (ie, no more solid excrement, but rather clear fluid). This took 3 hours on average. Between 200 g and 300 g of donor faeces was processed. Donor faeces were diluted in 500 mL of 0.9% saline solution and filtered through unfolded cotton gauzes. The 500 cc filtrate was used for transplantation, which was administered 2 hours after the last administration of Klean-Prep by duodenal tube in around 30 min using 50 cc syringes. After a short observation period, the patient was sent home.

### Hyperinsulinaemic euglycaemic clamp with stable isotope tracers and REE

Subjects were admitted at the metabolic unit after an overnight fast for a two-step hyperinsulinaemic euglycaemic clamp with stable isotope tracers,^13^ during which the rate of glucose disappearance (Rd) was measured to estimate insulin sensitivity. REE was determined using indirect calorimetry. A more comprehensive description of the clamp procedure and the calculation of isotope enrichments and endogenous glucose production (EGP) can be read in the online supplementary methods.

### Biochemistry

Fasting glucose, insulin, C reactive protein, lipopolysaccharide-binding protein, free fatty acids and lipid spectrum were determined using routine laboratory methods (see online supplementary methods). Faecal bile acids in 2×24 hour faeces were determined by gas chromatography as described earlier.^11^ Serum bile acids were extracted from plasma samples and measured by ultra-performance liquid chromatography-tandem mass spectrometry, as previously described.^12^ The 24-hour faecal energy content of lyophilised homogenised faeces (caloric bomb measurement) was determined with a bomb calorimeter (CBB 330, using as a standard benzoic acid 6320 cal/g). Faecal SCFAs were measured using high-performance liquid chromatography with ultraviolet detection as previously published.^14^ Chemokine ligand 2 (CCL2)) plasma levels were determined by the Human CCL2 Uncoated ELISA Kit (Invitrogen, ref# 88-7399-88) according to the manufacturer’s protocol.

### Quantitative PCR in subcutaneous adipose tissue biopsies

In fasted subjects, abdominal subcutaneous adipose tissue was aspirated using a hollow needle and a 50 cc syringe. RNA was isolated from adipose tissue biopsies using TriPure Isolation Reagent according to the manufacturer’s protocol (Roche, Germany). Complementary DNA (cDNA) was prepared using SensiFAST cDNA Synthesis Kit (Bioline, UK), and mRNA expression was measured via SensiFAST SYBR No-ROX Kit (Bioline). The primers for *MCP1*, *IL6*, *TNFα*, *IL10*, *Nf-κB*, *CD11b*, *CD68*, *IRS1* and *Leptin* are summarised in online supplementary table S3. Expression levels were normalised to *RPLP0*. We stained for the presence of macrophages (crown-like structures) in subcutaneous adipose tissue, but failed due to poor quality of the (needle biopsy achieved) adipose tissue samples.

10.1136/gutjnl-2019-318320.supp4 Supplementary data

### Intestinal transit time

Intestinal transit time was measured using Sitzmark capsules^15^ at baseline and 2 weeks after FMT. Capsules containing 24 plastic ring-markers each were ingested on days 1, 2 and 3 (72 markers in total), and then colon X-ray imaging was performed on day 4 to count the markers. The number of excreted markers was reported (72 minus the count). Reproducibility of the intestinal transit time measurement was determined by performing the Sitzmark capsule test twice (2 weeks apart) in 10 subjects with METS (online supplementary figure S1).

10.1136/gutjnl-2019-318320.supp5 Supplementary data

### Profiling of faecal microbiota composition by sequencing of the 16S rRNA gene

Faecal genomic DNA was isolated and faecal microbiota composition was profiled by sequencing the V4 region of the 16S rRNA gene. Preprocessing of the 16S rRNA amplicon sequence data resulted in a final data set that comprised 801 operational taxonomic units (OUPs) in 79 samples. A comprehensive description of the sequencing and preprocessing procedures can be read in the online supplementary methods.

### Metabolomics

Global targeted metabolite profiling analysis was carried out on fasting plasma samples by Metabolon (Durham, North Carolina), using ultra high-performance liquid chromatography coupled to tandem mass spectrometry, as previously described.^16^ Raw data were normalised to account for interday differences. Then, each biochemical was rescaled to set the median equal to 1. Missing values, generally due to the sample measurement falling below the limit of detection, were then imputed with the minimum observed value.

### Predictive modelling

Elastic net regularised classification models^17^ with stability selection^18^ were used to identify microbial predictors of effects on insulin sensitivity. Elastic net is a machine learning algorithm that selects features based on their relative importance in making a prediction (in this case, belonging to the METS-R or RYGB-R group). This model is especially suited when the number of samples is much smaller than the number of variables. Elastic net is described in more detail in the online supplementary methods.

### Power calculation, statistical analyses and exploratory analyses

We based our sample size calculation on the results of our earlier pilot FMT study,^10 11^ which showed a significant median increase in Rd on allogenic lean donor FMT. We anticipated that allogenic RYGB-D FMT to subjects with METS in our study would induce a similar overall increase in median Rd of 12 µmol/kg/min, while allogenic METS-D FMT would probably have less effect (5 µmol/kg/min) with an SD of 5 µmol/kg/min. Taking a 10% dropout rate into account, we would need 22 subjects with METS to be included overall. A non-Gaussian distribution for all clinical data was assumed, and thus results are presented as medians and IQRs. Statistical testing was carried out using (two-sided) non-parametric tests. For between-group comparisons, the unpaired Wilcoxon signed-rank test was used, whereas a paired test was used for within-group comparisons of repeated measurements. A false discovery rate-corrected p value below 0.05 was considered significant. For the exploratory analyses, we divided the RYGB-R group into responders (≥10% increase in Rd) and non-responders (<10% increase). The 10% is in line with our earlier studies that report 10% interindividual session variance between clamp days.^10 11 19^ The METS-R group was divided into deteriorators (≥10% decrease in Rd) and non-deteriorators (<10% decrease).

## Results

### Glucose metabolism: primary outcomes

The baseline characteristics of the subjects and donors are depicted in table 1. Figure 1A explains the study design. We observed a significant change in peripheral insulin sensitivity (mean Rd) on FMT from RYGB-D versus FMT from METS-D (figure 1B), our primary outcome parameter. When studying the groups separately, we observed that this differential effect was mainly driven by a significant decrease (median Rd from 40.6 to 34.0 µmol/kg/min, p<0.01) in the METS-R group (n=10), which received FMT from METS-D, while the increase in peripheral insulin sensitivity from 33.9 to 36.2 µmol/kg/min in the RYGB-R group (n=12), which were subjects with METS who received FMT from RYGB-D, was not statistically significant. Of note, baseline peripheral insulin sensitivity in RYGB-D was significantly higher than in METS-D (figure 1C). Finally, hepatic insulin sensitivity (suppression of EGP; figure 1D, E and online supplementary figure S2A) as well as the rate of lipolysis (online supplementary figure S2) were not significantly affected by either RYGB-D or METS-D FMT. Individual donor and recipient values for Rd and EGP, as well as response or deterioration status, can be found in online supplementary tables S2 and S3.

10.1136/gutjnl-2019-318320.supp6 Supplementary data

**Table 1:** Baseline characteristics

Variable	METS-R	RYGB-R	P value	METS-D	RYGB-D
Weight (kg)	119	119	0.99	118	114
BMI (kg/m²)	37.4	36.0	0.55	33.8	31.4
Fasting plasma glucose (mmol/L)	5.48	5.03	0.17		
Fasting insulin (pmol/L)	99.4	95.1	0.85	116	46
HOMA1-IR (mean)	3.14	2.89	0.65		
Rd (µmol/kg/min)	40.6	33.9	0.37	31.8	50.5
EGP suppression (%)	77.4	75.9	0.82	71.5	85.7
Lipolysis suppression (%)	67	66	0.86	64	74
Excreted Sitzmark (n)*	53	40	0.27	58	51
REE (kcal/kg)	17.5	17.4	0.84	18.5	17.3
Total cholesterol (mmol/L)	5.2	4.9	0.45	6.1	4.0
HDL (mmol/L)	1.18	1.06	0.17	1.08	1.60
Dietary intake					
Calories (kcal/day)	2033	1903	0.57	2073	2226
Fat (g/day)	73.0	67.9	0.64	66.3	82.7
Saturated fat (g/day)	29.6	25.1	0.28	23.6	30.0
Protein (g/day)	84.11	86.4	0.80	138.4	132.0
Carbohydrates (g/day)	214.6	206.2	0.80	139.1	193.4
Fibre (g/day)	16.71	19.0	0.22	17.3	18.9
Alcohol (units/week)	8	5	0.50	6	11

Means are reported unless stated otherwise.

*Median instead of mean reported.

BMI, body mass index; EGP, endogenous glucose production; FMT, faecal microbiota transplantation; HDL, high-density lipoprotein; HOMA1-IR, homeostatic model assessment-insulin resistance; METS-R, recipients with metabolic syndrome who received FMT from donors with metabolic syndrome; METS-D, metabolic syndrome donors; Rd, rate of glucose disappearance; REE, resting energy expenditure; RYGB-D, Roux-en-Y gastric bypass donor; RYGB-R, recipients with metabolic syndrome who received FMT from post-Roux-en-Y gastric bypass donors.

### Glucose metabolism in responders and deteriorators: exploratory analyses

At baseline, RYGB-D were significantly more insulin-sensitive than RYGB-R, whereas insulin sensitivity did not differ between METS-D and METS-R (figure 1F). Exploring the individual effects following FMT treatment (online supplementary figure S3A, B), we observed that Rd was not improved in any of the METS-R subjects. In contrast, Rd deteriorated in only one of the RYGB-R subjects (improvement or deterioration was defined as >10% increase or <10% decrease in Rd, respectively).

10.1136/gutjnl-2019-318320.supp7 Supplementary data

Moreover, there was no statistically significant correlation (Spearman’s rank) between change in Rd in the recipient and the difference in Rd between recipient and donor (online supplementary figure S3C, D). However, in the METS-R group, all subjects who deteriorated received FMT from a donor with poorer Rd (online supplementary figure S3C), while all subjects who did not change had received FMT from donors with similar or better Rd (online supplementary figure S3C, D).

### Changes in BMI REE and faecal energy excretion on FMT

BMI was not significantly different in METS-D versus METS-R (34 kg/m² in METS-D vs 37 kg/m² in METS-R, p=0.08). As expected, BMI in RYGB-R was higher (36 kg/m² vs 31 kg/m²) than in RYGB-D (p=0.023). BMI did not change significantly after FMT in either group at 2 or 8 weeks. REE did not change in either group (online supplementary figure S2C). Finally, faecal energy excretion (bomb calorimetry) did not change between week 0, 2 or 8 in either the RYGB-R (median from 1358 to 1021 calories per 48 hours between weeks 0 and 2, p=0.27) or the METS-R group (median from 1087 to 856 calories per 48 hours, p=0.37).

### Intestinal transit time, faecal bile acid concentrations and faecal SCFA after FMT

**Figure 2:**
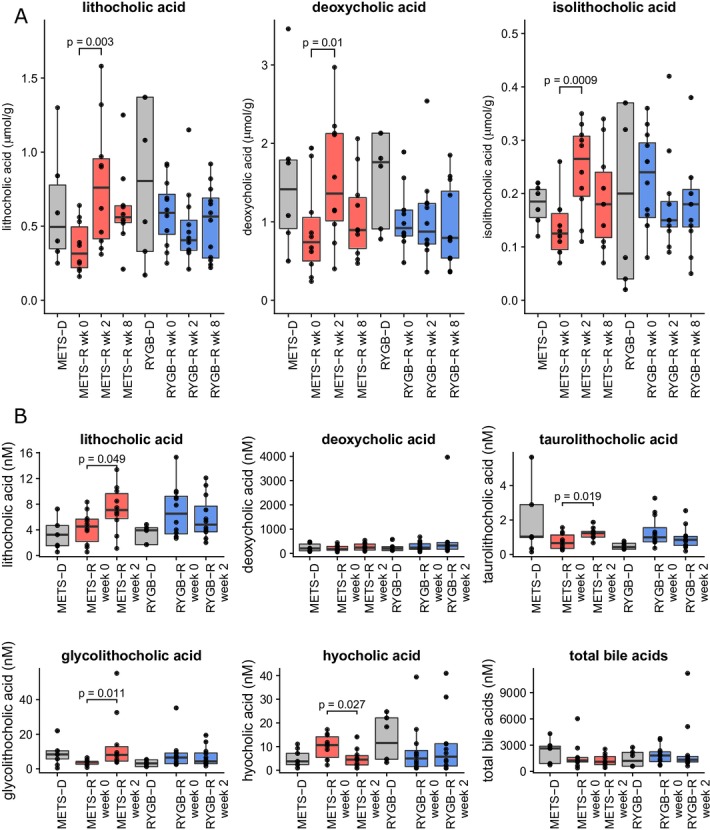
(A) Faecal bile acid content at weeks 0, 2 and 8 for recipients and donors at baseline for isolithocholic acid, lithocholic acid and deoxycholic acid. (B) Serum bile acid content for lithocholic acid, deoxycholic acid, taurolithocholic acid, glycolithocholic acid, hyocholic acid and total bile acids. Bile acids that were not significantly affected in serum are shown in online supplementary figure S3. Data are presented as median±IQR in µmol/g dry faeces. Individual points represent individual subjects. For comparison between the groups, a Student’s t-test was used if the bile acid was normally distributed. If not, a Wilcoxon signed-rank test was used. All p values <0.1 are reported in the figures. METS-D, metabolic syndrome donor; METS-R, metabolic syndrome recipient; RYGB-D, Roux-en-Y gastric bypass donor; RYGB-R, Roux-en-Y gastric bypass recipient.

To verify the used Sitzmark method for intestinal transit time specifically for our study group, we first performed the test twice (n=10 subjects repeated 2 weeks apart) in our subjects with METS in order to look at reproducibility. As depicted in online supplementary figure S1 (Bland-Altman plot), our Sitzmark intestinal transit time calculation was reproducible (90% of measurement pairs within coefficient of variation of 2 SD). Intestinal transit did not change in the METS-R group (from 53±11 to 49±10 excreted markers/72 hours; figure 1G and online supplementary figure S2D), whereas in the RYGB-R group we observed a trend towards faster intestinal transit (from 39±18 to 56±12 excreted markers/72 hours; p=0.052) (figure 1H and online supplementary figure S2D). Individual donor and patient transit time can also be found in online supplementary tables S2 and S3. Because microbially produced secondary bile acids and SCFA have been demonstrated to regulate intestinal transit in mouse models,^20^ we next analysed their levels. Between baseline and 2 weeks after FMT, we observed a significant increase in faecal levels of lithocholic acid, isolithocholic acid and deoxycholic acid (p<0.01) in the METS-R group, whereas no effect was seen in the RYGB-R group (figure 2A). Faecal cholic acid, chenodeoxycholic acid and ursodeoxycholic acid did not change significantly in either group (data not shown). In agreement with the faecal levels, we also observed that the levels of lithocholic acid were increased in fasting plasma samples of the METS-R group (p=0.049), whereas deoxycholic acid plasma levels were unchanged. Although total plasma bile acids concentrations were unchanged in both groups, fasting plasma taurolithocholic acid (p=0.019) and glycolithocholic acid levels (p=0.011) were increased in the METS-R group, whereas plasma hyocholic acid was decreased (p=0.027) (figure 2B). Other plasma bile acids were unchanged in either group (online supplementary figure S4). Finally, total faecal SCFA propionate and butyrate levels (but not acetate) increased significantly in the METS-R group at week 2 but returned to baseline at week 8 (online supplementary figure S5).

10.1136/gutjnl-2019-318320.supp8 Supplementary data

10.1136/gutjnl-2019-318320.supp9 Supplementary data

### Subcutaneous adipose tissue inflammation after FMT

**Figure 3:**
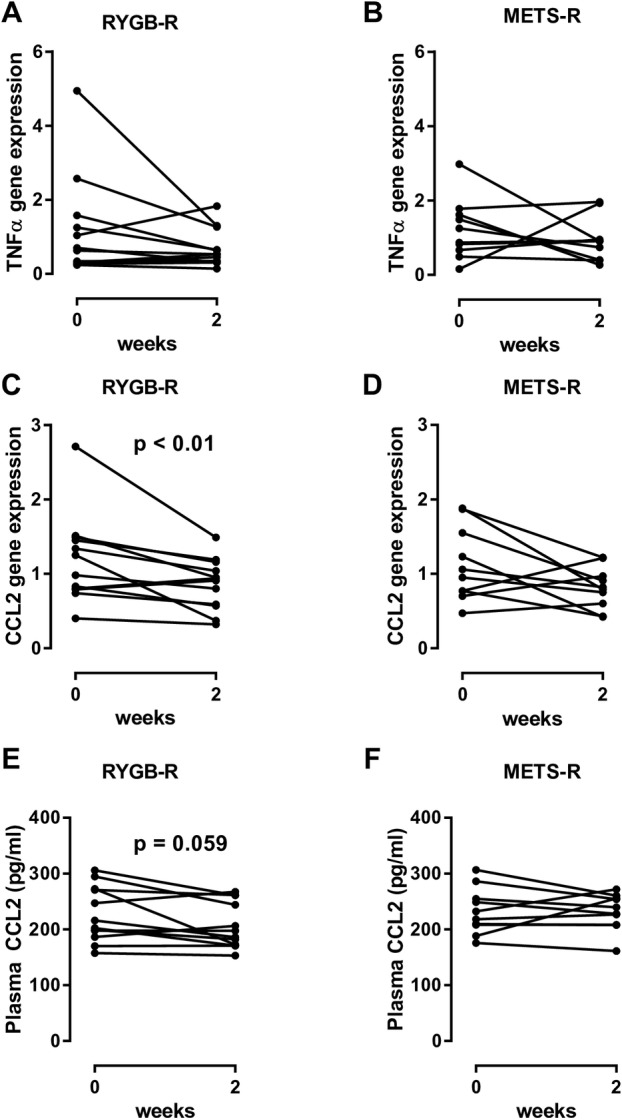
(A and B) Change in TNF-α gene expression in both groups. (C and D) Change in CCL2 gene expression in subcutaneous adipose tissue between weeks 0 and 2 in both groups. (E and F) Plasma CCL2, measured by ELISA. Each line represents an individual subject. For comparison between baseline and week 2, a paired Wilcoxon signed-rank test was used. CCL2, chemokine ligand 2; FMT, faecal microbiota transplantation; METS-R, recipients with metabolic syndrome who received FMT from donors with metabolic syndrome; RYGB-R, recipients with metabolic syndrome who received FMT from post-Roux-en-Y gastric bypass donors; TNF-α, tumour necrosis factor  alpha.

While expression of most of the genes in our panel of inflammatory genes was largely unaffected (figure 3A, B shows tumour necrosis factor alpha, the rest not shown), expression of CCL2 in subcutaneous adipose tissue was significantly reduced (figure 3C; p=0.01) in biopsies of the RYGB-R but not in the METS-R group (figure 3D). We subsequently found that fasting plasma CCL2 levels were also reduced in the RYGB-R group (p=0.059; figure 3E) but not in the METS-R group (figure 3F).

### Gut microbiota

**Figure 4:**
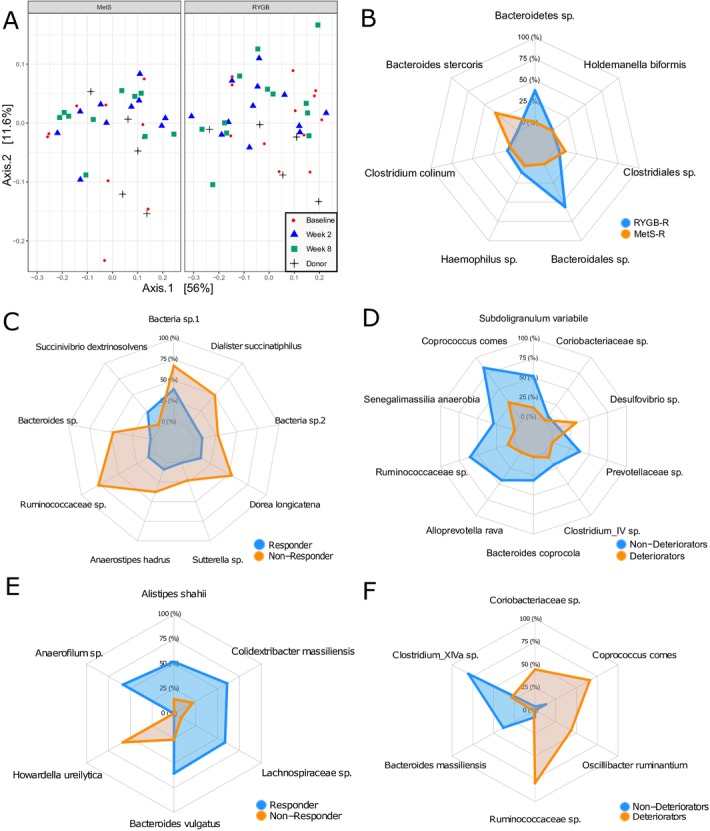
(A) Principal component analysis plot of weighted UniFrac distances of microbiota composition at baseline, week 2 and week 8 and donors. The left panel shows METS-R and METS-D, and the right panel shows RYGB-R and RYGB-D. (B) Spider plot showing operational taxonomic unit (OTUs) of which relative change best discriminated RYGB-R subjects from METS-R subjects. (C) Spider plot of OTUs of which the relative change best discriminated RYGB-R responders from RYGB-R non-responders. (D) Spider plot of OTUs of which the relative change best discriminated METS-R deteriorators from METS-R non-deteriorators. (E) Spider plot of OTUs of which baseline abundance best discriminated responders from non-responders and (F) OTUs of which baseline abundance best discriminated deteriorators from non-deteriorators. METS-D, metabolic syndrome donors; METS-R, metabolic syndrome recipient; RYGB-D, Roux-en-Y gastric bypass donor; RYGB-R, Roux-en-Y gastric bypass recipient.

As depicted in figure 4A (principle component analysisplot), using conventional statistics allogenic FMT with either METS-D or RYGB-D had only moderate effects on faecal microbiota composition. In line, faecal microbial diversity (Shannon index) before and after FMT did not differ in either group, between groups, between donors and recipients at baseline, or between responders/non-responders or deteriorators/non-deteriorators (online supplementary figure S6). Using predictive modelling, we have identified several microbiota that changed differentially between treatment groups. These are depicted in figure 4B.

10.1136/gutjnl-2019-318320.supp10 Supplementary data

As we previously observed differential individual recipient responses to donor FMT,^11^ we next determined whether there was an association between baseline microbiota composition as well as with compositional *change* (delta microbiota abundance) on one hand and clinical response to FMT on the other. To this end, we divided the RYGB-R group into responders (n=5) and non-responders (n=7), and the METS-R group into deteriorators (n=6) and non-deteriorators (n=4). Response was defined as ≥10% increase in Rd, while deterioration was defined as ≥10% decrease in Rd. Using predictive modelling (elastic net), we identified OTUs of which the relative change was predictive of responder/non-responder status in RYGB-R (figure 4C) and deteriorator/non-deteriorator status in METS-R (figure 4D). Also we observed OTUs of which baseline abundance was predictive of recipient response status (figure 4E for RYGB-R and figure 4F for METS-R). We found that *Alistipes shahii* was predictive of responder status at baseline (figure 4E) in our predictive model and was significantly more abundant in responders versus non-responders (p=0.009, Wilcoxon; online supplementary figure S7A). Second, change (increase) in *Anaerostipes hadrus*, a butyrate producer that is associated with caloric restriction,^21^ was strongly associated with responder status (figure 4C); its abundance tripled in the responders, while it remained stable in the non-responders (p=0.051; online supplementary figure S7C). Third, increase in *Desulfovibrio* ssp was predictive of metabolic deterioration on allogenic METS-D FMT (figure 4D and online supplementary figure S7D), which is notable as this strain is known to be increased in faeces of patients with T2D.^22^ Finally, high *Coprococcus comes* abundance at baseline was predictive of deteriorator status (figure 4F and online supplementary figure S7B), while an increase in *C. comes* was predictive of non-deteriorator status (figure 4D and online supplementary figure S7E), and a linear correlation between change in Rd and change in *C. comes* abundance was observed (online supplementary figure S7F).

10.1136/gutjnl-2019-318320.supp11 Supplementary data

**Figure 5:**
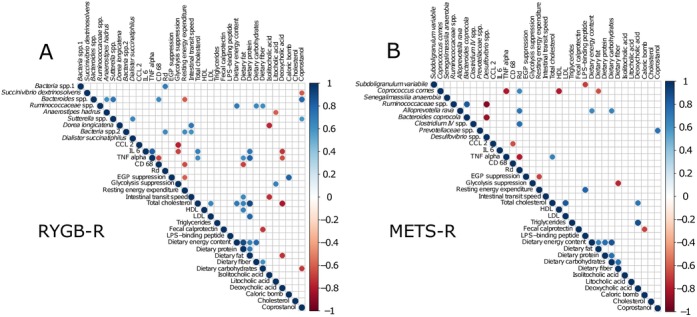
(A and B) Correlation graphs of changes between weeks 0 and 2 in relevant clinical variables and faecal microbiota operational taxonomic unit (OTUs) for both groups. A shows RYGB-R and B shows METS-R. Only significant (Spearman) correlations with p<0.05 are shown. These correlations have not been corrected for false discovery. Colours indicate increase (blue) or decrease (red). CCL2, chemokine ligand 2; CD68, cluster of differentiation 68; EGP suppression, suppression of endogenous glucose production by insulin (hepatic insulin sensitivity); HDL, high-density lipoprotein; IL 6, interleukin 6; LDL, low-density lipoprotein; LPS, lipopolysaccharide; METS-R, metabolic syndrome recipient; Rd, rate of glucose disappearance (peripheral insulin sensitivity); RYGB-R, Roux-en-Y gastric bypass recipient;  TNF alpha, tumour necrosis factor alpha.

As shown in figure 5A, B, we correlated the abundance of microbial taxa with metabolic and anthropometric parameters in the RYGB-R and METS-R groups. We observed that changes in intestinal transit positively correlated with changes in the relative abundance of *Dorea longicatena* in the RYGB-R group (r=0.72, p=0.01) (online supplementary figure S7G). As with *C. comes,* changes in peripheral insulin sensitivity (Rd) also correlated positively with changes in the relative abundance of *Alloprevotella rava* (r=0.72, p=0.024; online supplementary figure S7H) and an OTU from *Clostridium cluster IV* in the METS-R group (r=0.77, p=0.009; online supplementary figure S7I).

### Metabolomics

**Figure 6:**
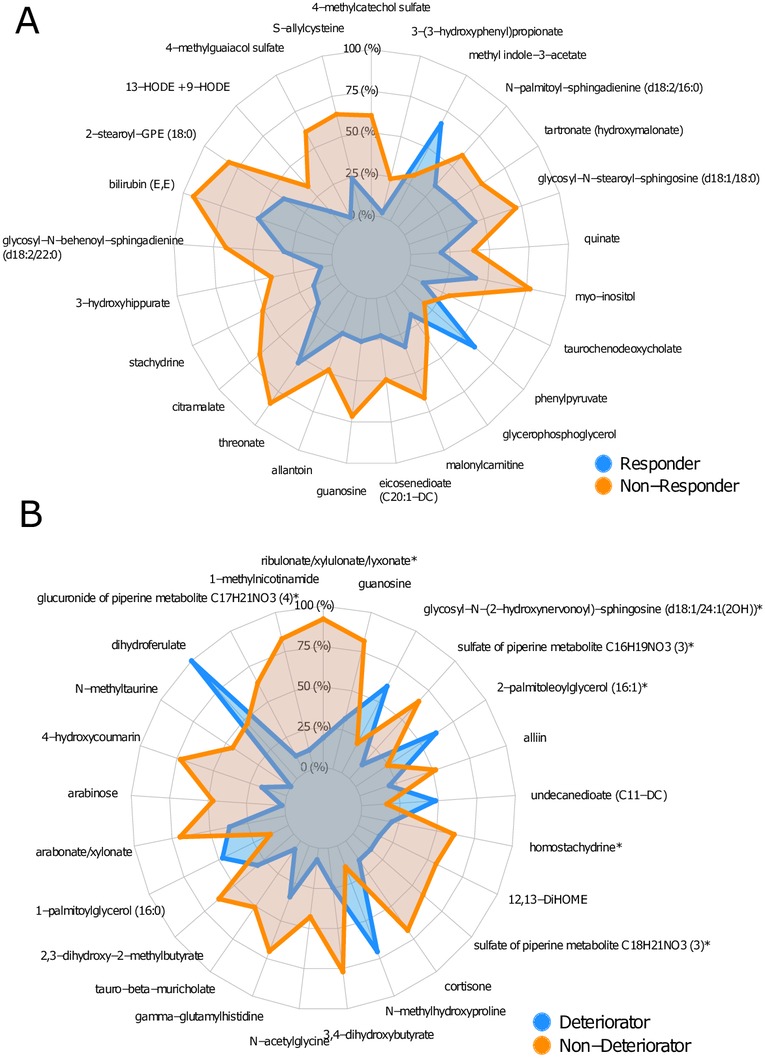
(A) Spider plot of top 25 metabolites of which the change in relative abundance best differentiated between responders and non-responders (in RYGB-R). (B) Spider plot of top 25 metabolites of which the change in relative abundance best differentiated between deteriorators and non-deteriorators (in METS-R). GPE, glycerophosphoethanolamine; HODE, Hydroxyoctadecadienoic acid; METS-R, metabolic syndrome recipient; RYGB-R, Roux-en-Y gastric bypass recipient.

We used predictive modelling to identify fasting plasma metabolites that differentiate between treatment groups (METS-R vs RYGB-R), between responders versus non-responders, and deteriorators versus non-deteriorators. In the responders versus non-responders analyses, a number of metabolites were differentially changed between baseline and 2 weeks, including methyl indole-3-acetate and phenylpyruvate (figure 6A). Similarly, a number of plasma metabolites differentiated between deteriorators and non-deteriorators, including plasma levels of dihydroferulate (figure 6B). Finally, a number of metabolites in baseline fasting plasma samples predicted metabolic response versus non-response (online supplementary figure S8A), and metabolic deterioration versus non-deterioration (online supplementary figure S8B). In this regard, the plasma 4-hydroxyphenylpyruvate was among others associated with response, whereas plasma sphingomyelin subsets were associated with deterioration in insulin sensitivity (online supplementary figure S8A, B).

10.1136/gutjnl-2019-318320.supp12 Supplementary data

## Discussion

Over the past years the intestinal microbiota composition has been associated with altered metabolic processes,^23^ yet data on causality in human metabolism are scarce. In accordance with our previous FMT studies,^10 11^ we show in this study that individual FMT donor characteristics can significantly influence metabolic effects of FMT in the recipients. We found that METS-D FMT had a negative effect on Rd, while RYGB-D FMT mainly accelerated intestinal transit. As our METS FMT donors were characterised by lower peripheral insulin sensitivity (Rd) than their METS recipients, a gut microbiota-driven transmissible trait of insulin resistance in obesity is a tempting explanation. Although the sample size of our study was small, our data do underscore the potential causal effect of individual FMT gut microbiome composition on recipients’ metabolism.

### Metabolic traits of allogenic FMT donors may drive recipient metabolic response

Similar to our previous FMT studies,^10 11^ we found that donor FMT treatment affects faecal microbiota composition and has a transient and differential metabolic effect in insulin-resistant subjects. With respect to metabolic phenotype, it is interesting to note that allogenic METS-D FMT resulted in a significant decline in Rd, while RYGB-D FMT did not reduce peripheral insulin sensitivity (Rd) in the recipients. However, the clinical effect in our human subjects was much smaller and more diverse than the large metabolic improvements that were reported in DIO mice receiving post-RYGB FMT treatment.^2^ Interestingly, we previously observed similar effects on CCL2 on changes in gut microbiota composition in mice,^24^ suggesting regulation of adipose tissue inflammation by gut microbiota in humans. Moreover and in line with our previous work,^10^ our RYGB-R non-responders all had a low Rd at baseline, suggesting that when insulin sensitivity is already poor residual metabolic flexibility is insufficient and less effect from microbial intervention can be expected, which may explain the lack of significant improvement in the RYGB-R group.

### FMT affects bile acids, SCFAs, metabolites and intestinal transit time

Animal studies have shown that gut microbiota composition affects bile acid metabolism.^25^ In contrast to lean healthy donor FMT that specifically increased the primary bile acid cholic acid concentrations,^11 26^ in the current study we observed a significant increase of the secondary bile acid lithocholic acid in plasma and faeces, and of isolithocholic acid and deoxycholic acid in faeces of METS-R (p<0.01). Underscoring potential clinical and therapeutic relevance, previous studies have shown that bile acid concentrations are altered in patients with T2D,^27 28^ and treatment with bile acid sequestrants affects glucose metabolism.^29^ Moreover, bile acids are thought to increase intestinal transit through takeda G protein-coupled receptor 5 (TGR5),^30^ which is in line with our finding that transit time correlated inversely with faecal isolithocholic acid (figure 5A). The observed increase in secondary bile acids in METS-R, which coincided with higher bile acid levels in the donors, may indicate increased transport of bile acids into the colon induced by higher microbial bile salt hydrolase activity. Finally, with respect to changes in fasting plasma metabolites (see figure 6A), methyl indole-3-acetate has been linked to an improved metabolic profile in a murine model of insulin resistance.^31^ In line, older literature has linked phenylpyruvate to increased insulin secretion from beta cells,^32^ which may explain the improved insulin sensitivity in the responder patients. In contrast, dihydroferulate (see figure 6B) was associated with worsening of insulin resistance. Interestingly, dihydroferulate is also known as 3-(4-hydroxy-3-3methoxyphenyl) propionic acid, which is a derivative from propionic acid, an SCFA that has recently been linked to increased risk of T2D.^33^

### Gut microbiota composition at baseline may predict metabolic effects of FMT

By employing predictive modelling using machine learning, we have identified several microbial OTUs of interest that may predict metabolic response. In this regard *A. shahii* is associated with altered cardiometabolic status.^34^ Also, *Clostridium cluster IV* has been linked to healthy glucose metabolism in humans.^35^ In conjunction, *D. longicatena* (previously linked with a healthy gut microbiota homeostasis^36 37^ and with more favourable metabolic outcomes in insulin-resistant obese subjects^11 38^) predicted FMT response and was associated with increased intestinal transit time. Subsequently, whereas baseline plasma levels 4-hydroxyphenylpyruvate were among others associated with beneficial response on FMT, plasma sphingomyelin levels known to drive insulin resistance^39^ were indeed associated with deterioration in insulin sensitivity (online supplementary figure S8A, B). Together, these data suggest that gut microbiota-derived plasma metabolites indeed may regulate insulin resistance balance in obese humans.

### Use of allogenic donors with METS for the control group

In our previous studies autologous faecal transplantation was done in the control group. However, in theory the observed effects after allogenic versus autologous FMT may stem from a non-specific immunological reaction on receiving foreign microbiota rather than from a specific microbiota composition. This key issue was addressed in the current study by using an allogenic control group. Our previous studies show that metabolic effects and changes in microbiota composition are temporary and have resolved 12–18 weeks after FMT,^10 11^ so we did not expect lasting negative metabolic effects. Indeed, our results show homeostatic model assessment-insulin resistance (HOMA-IR) at 8 weeks had not deteriorated in our METS-R control group (slight improvement, p=0.34) in comparison with HOMA-IR at baseline. From the current data we conclude that the use of allogenic METS control donors in future studies seems unnecessary and undesirable, resolving a fundamental methodological issue in human FMT studies in subjects with METS.

### Limitations

Our study has several limitations. First, the sample size of our study was small and the metabolic effects were moderate. Nevertheless, our clinical data suggest that the metabolic signature of the FMT donor can be transferred temporarily to the recipient and that this may be driven by specific intestinal microbial signatures.

Second, subjects were allowed to keep their own diet, which was closely monitored by dietary recall, but allows for more variation in the data compared with a standardised diet.^40^ Only European men of Caucasian descent were included, and thus results may not apply to the general multiethnic Western world population, subsets of which have different faecal microbiota composition.^41^

Third, a possible explanation for a lack of effect of the RYGB-D FMT is the fact that the RYGB microbiota is well selected and kept under pressure by the rearrangements of the GI tract introduced by bariatric surgery. In the absence of such rearrangements in intestinal physiology, the RYGB-adapted microbiota may lack the selective force for the maintenance of its structure, and therefore cannot be engrafted durably in the recipient human host. This could explain the difference with the observed beneficial effects in the murine RYGB FMT model^2^ as these mice were kept in extremely controlled conditions and isolated from the external environment, so that environmental microbes and possible normal gut colonisers are not encountered. On the other hand, it is also likely that the human host immune system develops resilience, which in combination with the persistent adherence to one’s own lifestyle including diet^40^ could explain the return of intestinal microbiota composition to the baseline situation and the modest magnitude of short-term and long-term metabolic response.^42^ Nevertheless, our data underscore the potential causal effect of individual gut microbiome composition on human metabolism. Future FMT studies will have to confirm our findings, but additional prospective studies are required to further unravel the potential interaction between dietary intake, intestinal microbiota composition and subsequent changes in intestinal and whole body metabolism.

10.1136/gutjnl-2019-318320.supp1 Supplementary data

10.1136/gutjnl-2019-318320.supp2 Supplementary data
