# Supplementary material for: Donor metabolic characteristics drive effects of faecal microbiota transplantation on recipient insulin sensitivity, energy expenditure and intestinal transit time
Source: Gut. 2019 May 30;69(3):502–12. doi: 10.1136/gutjnl-2019-318320 (PMC7034343; doi:10.1136/gutjnl-2019-318320)
Supplement: Supplementary data [file gutjnl-2019-318320supp003.pdf]

Supplementary methods

Donor screening

All donors completed questionnaires regarding dietary and bowel habits, travel history, comorbidity including (family history of) diabetes mellitus and medication use. Donors were screened for the presence of infectious diseases as previously published[1]. Donors with complaints of irritable bowel syndrome or colon cancer in a first-degree relative before the age of 60 were also excluded as they diseases have been linked to the gut microbiota.

Donor blood was screened for presence of (antibodies to) human immunodeficiency virus; human T-lymphotropic virus; Hepatitis A, B, and C; cytomegalovirus (CMV); Epstein–Barr virus (EBV); strongyloides; amoebiasis and lees. Presence of infection resulted in exclusion, although previous, non-active infections with EBV and CMV were included. Donors were also excluded if screening of their feces revealed the presence of pathogenic parasites (e.g. blastocystis hominis, dientamoeba fragilis, giardia lamblia), bacteria (*Shigella*, *Campylobacter*, *Yersinia*, *Salmonella, enteropathogenic E. coli* and *Clostridium difficile*) or viruses (noro-, rota-, astro-, adeno (40/41/52)-, entero-, parecho- and sapovirus).

Biochemistry

Fasting glucose (Hitachi), insulin (Diagnostic products), C-reactive protein (CRP, Roche, Switzerland), free fatty acids (Wako) and lipopolysaccharide binding protein (LBP, HyCult) were determined in fasted plasma samples. Total cholesterol, high density lipoprotein cholesterol (HDLc) and triglycerides (TG) were determined in EDTA-containing plasma using commercially available enzymatic assays (Randox, Antrim, UK and DiaSys). All analyses were performed using a Selectra autoanalyzer (Sopachem, The Netherlands). Low density lipoprotein cholesterol (LDLc) was calculated using the Friedewald formula. FFA concentrations were determined with an enzymatic colorimetric method (NEFA-C test kit; Wako Chemicals, Neuss, Germany). Insulin was determined on an Immulite 2000 system (Diagnostic Products, Los Angeles, CA, USA).

Hyperinsulinaemic euglycemic clamp with stable isotope tracers and resting energy expenditure

Subjects were admitted at the metabolic unit after an overnight fast for the metabolic flux measurements [2]. Resting energy expenditure (REE) using indirect calorimetry was determined and a catheter was inserted into an antecubital vein for infusion of stable-isotope tracers, insulin, and glucose. Another catheter was inserted into a contralateral hand vein and kept in a thermoregulated (60°C) clear plastic box for sampling of arterialized venous blood. Saline was infused as 0.9% NaCl at a rate of 50 mL/h to keep the catheters patent. [6,6-$^{2}H_{2}$]glucose and [1,1,2,3,3-$^{2}H_{5}$]glycerol were used as tracers (>99% enriched; Cambridge Isotopes, Andover, MA) to study glucose kinetics and lipolysis (total triglyceride hydrolysis). The exact methodology of the two-step hyperinsulinaemic euglycaemic clamp and the calculation of isotope enrichments and endogenous glucose production (EGP) can be read in the supplementary methods.

At $t = 0$ h, blood samples were drawn for determination of background enrichments. Then, a primed continuous infusion of isotopes was started: [6,6-$^{2}H_{2}$]glucose (prime: 8.8 μmol/kg; continuous: 0.11 μmol · kg^{-1} · min^{-1}), [1,1,2,3,3-$^{2}H_{5}$]glycerol (prime: 1.6 μmol/kg; continuous: 0.11 μmol · kg^{-1} · min^{-1}) and continued until the end of the study. After a 2-h equilibration period (14 h of fasting), three blood samples were drawn for isotope enrichments and one sample for glucoregulatory hormones and free fatty acids (FFA). Thereafter ($t = 3.0$ h), a 2-step hyperinsulinemic euglycemic clamp was started: step one included an infusion of insulin at a rate of 20 mU · m^{-2} · min^{-1} (Actrapid 100 IU/mL; Novo Nordisk Farma BV, Alphen a/d Rijn, Netherlands) to assess hepatic insulin sensitivity. Glucose 20% was started to maintain a plasma glucose concentration of 5 mmol/L. [6,6-$^{2}H_{2}$]Glucose was added to the 20% glucose solution to achieve glucose enrichments of 1% to approximate the values for enrichment reached in plasma and thereby minimizing changes in isotopic enrichment due to changes in the infusion rate of exogenous glucose[3]. Plasma glucose concentrations were measured every five minutes at the bedside. Five blood samples were drawn at five minute intervals for the measurement of glucose concentrations and isotopic enrichments. Another blood sample was drawn for measurement of glucoregulatory hormones and FFA. Hereafter, insulin infusion was increased to a rate of 60 mU · m^{-2} · min^{-1} (step 2) to assess peripheral insulin sensitivity. After another 2 h ($t = 7$ h), blood sampling was repeated. Endogenous glucose production (EGP) and the peripheral glucose uptake of glucose (Rd) were calculated by using modified versions of the

Steele Equations, as described previously[3]. EGP and Rd were expressed as μmol · kg^{-1} · min^{-1}. Total triglyceride hydrolysis/lipolysis (glycerol turnover) was calculated by using formulas for steady state kinetics adapted for stable isotopes and was expressed as μmol · kg^{-1} · min^{-1}[4].

Profiling of fecal microbiota composition by sequencing of the 16S rRNA gene

Fecal genomic DNA was isolated from 100 mg of feces using repeated bead beating and purification in the QIAcube (QIAGEN) similar to what previously described, with bead beating at 5.0 m/s for 60 s in a FastPrep®-24 instrument (MP Biomedicals)[5]. Fecal microbiota composition was profiled by sequencing the V4 region of the 16S rRNA gene on an Illumina MiSeq instrument (llumina RTA v1.17.28; MCS v2.5) with 515F and 806R primers designed for dual indexing[6] and the V2 Illumina kit (2x250 bp paired-end reads). 16S rRNA genes were amplified in duplicate reactions as previously described[5].

Pre-processing of 16S rRNA amplicon sequence data

Raw fastq files were quality controlled using FastQC (version 0.11.5). Illumina paired-end reads were processed using the Mothur pipeline (version 1.39.5). After merging forward and reverse reads, contigs were screened to ensure absence of ambiguous bases and a length between 252 and 254 bases. Contigs were aligned to the Silva reference (version 128), dereplicated and subsequently preclustered (allowing a maximum of two differences). Singletons (sequences with an abundance of one in the entire dataset) were removed. The remaining sequences were chimera-filtered with chimera.vsearch and classified using the Silva taxonomy reference (version 128) with a 80% confidence cutoff. Sequences classified as mitochondria, chloroplasts, eukaryota, as well as un-classified sequences were removed. Remaining sequences were clustered using the Opticlust distance-based algorithm to 97% OTUs. A phylogenetic tree was constructed with the “double-precision” build of FastTree 2.1 using the abundance-based representative sequences of the OTUs[7]. The resulting OTU table was rarefied to 60 000 counts per sample using the phyloseq package in R. OTUs with a mean relative abundance of less than 0.001% (i.e. a mean of less than one count per two samples) were filtered out. The final dataset was comprised of 801 OTUs in 79 samples.

Machine learning models

Elastic Net regularized classification models [8] with stability selection [9] were implemented in Python 2.7 (www.python.org) as a feature selection tool. In this paper we used the elastic net model which has been widely used for performing feature selection in biological data[10,11]. Elastic net is an embedded sparsity inducing feature selection approach, which combines the sparse regularization and stability selection procedures. This method is a regularization-based method which combines the advantages of two methods such as lasso and ridge regression[8]. Its L2-norm based shrinkage encourages highly correlated features to have similar weights, whereas L1-norm based shrinkage encourages a sparse solution.

Input data (i.e. OTU predictors) were used to predict class membership. For each analysis, input OTUs were filtered to a minimum abundance of at least 10 counts per sample in respective subset of subjects. To train each model, the two hyperparameters (the alpha – the size of the regularization penalty, and the L1 ratio – the ration of L1-norm / L2-norm in the model penalty) were optimized using a 4-fold Cross-Validation procedure on a subset comprising 80% of the data. The model was then tested on the remaining 20% of the data not used in the training. This procedure was repeated 100 times per analysis, each time using different random splittings of the data into train and test subsets. The stability of each feature was calculated as the number of times (out of 100) the respective feature was kept by the model (i.e. the number of times out of 100 runs that the feature had a non-zero regression coefficient). A minimum stability threshold of 60% was applied to all selected features.

References

1 van Nood E, Vrieze A, Nieuwdorp M, *et al.* Duodenal infusion of donor feces for recurrent Clostridium difficile. *N Engl J Med* 2013;**368**:407–15. doi:10.1056/NEJMoa1205037

2 Soeters MR, Lammers NM, Dubbelhuis PF, *et al.* Intermittent fasting does not affect whole-body glucose, lipid, or protein metabolism. *Am J Clin Nutr* 2009;**90**:1244–51. doi:10.3945/ajcn.2008.27327

3 Finegood DT, Bergman RN, Vranic M. Estimation of endogenous glucose production during hyperinsulinemic-euglycemic glucose clamps. Comparison of unlabeled and labeled exogenous glucose infusates. *Diabetes* 1987;**36**:914–24.

4 Ackermans MT, Ruiter AF, Endert E. Determination of glycerol concentrations and glycerol isotopic enrichments in human plasma by gas chromatography/mass spectrometry. *Anal Biochem* 1998;**258**:80–6. doi:10.1006/abio.1997.2535

5 Mobini R, Tremaroli V, Stahlman M, *et al.* Metabolic effects of Lactobacillus reuteri DSM 17938 in people with type 2 diabetes: A randomized controlled trial. *Diabetes Obes Metab* 2017;**19**:579–89. doi:10.1111/dom.12861

6 Kozich JJ, Westcott SL, Baxter NT, *et al.* Development of a dual-index sequencing strategy and curation pipeline for analyzing amplicon sequence data on the MiSeq Illumina sequencing platform. *Appl Environ Microbiol* 2013;**79**:5112–20. doi:10.1128/AEM.01043-13

7 Price MN, Dehal PS, Arkin AP. FastTree 2 – Approximately Maximum-Likelihood Trees for Large Alignments. *PLoS One* 2010;**5**:e9490. doi:10.1371/journal.pone.0009490

8 Zou H, Hastie T. Regularization and variable selection via the elastic net. *J R Stat Soc Ser B (Statistical Methodol* 2005;**67**:301–20. doi:10.1111/j.1467-9868.2005.00503.x

9 Meinshausen N, Bühlmann P. Stability selection. *J R Stat Soc Ser B (Statistical Methodol* 2010;**72**:417–73. doi:10.1111/j.1467-9868.2010.00740.x

10 Das J, Gayvert KM, Bunea F, *et al.* ENCAPP: elastic-net-based prognosis prediction and biomarker discovery for human cancers. *BMC Genomics* 2015;**16**:263. doi:10.1186/s12864-015-1465-9

11 Saeys Y, Inza I, Larrañaga P. A review of feature selection techniques in bioinformatics. *Bioinformatics* 2007;**23**:2507–17. doi:10.1093/bioinformatics/btm344
