# Supplementary material for: Donor metabolic characteristics drive effects of faecal microbiota transplantation on recipient insulin sensitivity, energy expenditure and intestinal transit time
Source: Gut. 2019 May 30;69(3):502–12. doi: 10.1136/gutjnl-2019-318320 (PMC7034343; doi:10.1136/gutjnl-2019-318320)
Supplement: Supplementary data [file gutjnl-2019-318320supp004.pdf]

Supplemental Table 3
Primers used for mRNA expression analysis

Gene	*Forward*	*Reverse*
36B4	ACG GGT ACA AAC GAG TCC TG	GCC TTG ACC TTT TCA GCA AG
MCP1	TGT CCC AAA GAA GCT GTG ATC	ATT CTT GGG TTG TGG AGT GAG
TNFa	CAG CCT CTT CTC CTT CCT GAT	GCC AGA GGG CTG ATT AGA GA
CD11b	ACT TGC AGT GAG AAC ACG TAT G	TCA TCC GCC GAA AGT CAT GTG
IL-10	GAT GCC TTC AGC AGA GTG AA	GCA ACC CAG GTA ACC CTT AAA
NFkB	GCA CCC TGA CCT TGC CTA TT	GCT CTT TTT CCC GAT CTC CCA
CD68	CCC CAA CAA AAC CAA GGT CC	GGA GGT CCT GCA TGA ATC CAA A
IL-6	AGG CAC TGG CAG AAA ACA AC	TTT TCA CCA GGC AAG TC TCC
IRS1	TAT GCC AGC ATC AGT TTC CA	TTT GCT GAG GTC ATT TAG GTC TT
Leptin	TTC ACA CAC GCA GTC AGT CT	CTG CCA GTG TCT GGT CCA TC
